# Supplementary material for: Factors associated with unawareness of HIV-positive status in urban Ethiopia: Evidence from the Ethiopia population-based HIV impact assessment 2017-2018
Source: PLoS One. 2021 Aug 11;16(8):e0255163. doi: 10.1371/journal.pone.0255163 (PMC8357455; doi:10.1371/journal.pone.0255163)
Supplement: S1 File — (DOCX) [file pone.0255163.s001.docx]

**S1 File**

**The EPHIA Study Team**

| Yimam Getaneh (PI)  Saro Abdella  Wudinesh Belete  Tsigereda Kifle  Abebe Habteselassie  Minilik Demissie  G/medhin G/Michael  Habtamu Teklie  Ebba Abate | Ethiopian Public Health Institute |
| --- | --- |
| Eleni Seyoum | Federal HIV/AIDS Prevention and Control Office |
| Esayas Muleta | Central Statistics Agency |
| Frehywot Eshetu  Jelaludin Ahmed  Clare Dykewicz  Ashenafi Haile  Yared Tedla  Jeff Hanson  Christine Ross  Biniyam Eskinder  Solomon Fekadie  Wondimu Teferi | U.S Centers for Disease Control and Prevention, Ethiopia |
| Drew Voetsch (PI)  Aderonke S. Ajiboye  Sehin Birhanu  Kristin Brown  Edith Nyangoma  Bharat Parekh  Hetal Pate  Christine W. West | U.S Centers for Disease Control and Prevention, Atlanta |
| Sileshi Lulseged (PI)  Zenebe Melaku  Halegnaw Eshete  Terefe Gelibo  Belete Tegbaru  Yohanes Demissie  Nadew Tademe | ICAP-Columbia University, Ethiopia |
| Jessica Justman (PI)  David Hoos  Mansoor Farahani  Karampreet Sachathep  Suzue Saito  Andrea Low  Chelsea Solmo | ICAP-Columbia University-New York |
