# Supplementary material for: Factors associated with unawareness of HIV-positive status in urban Ethiopia: Evidence from the Ethiopia population-based HIV impact assessment 2017-2018
Source: PLoS One. 2021 Aug 11;16(8):e0255163. doi: 10.1371/journal.pone.0255163 (PMC8357455; doi:10.1371/journal.pone.0255163)
Supplement: Attachment — Submitted filename: Rebuttal Letter.docx [file pone.0255163.s002.docx]

PONE-D-21-05302
Factors associated with Unawareness of HIV Positive Status in Urban Ethiopia: Evidence from the Ethiopia Population-based HIV Impact Assessment 2017-2018
PLOS ONE

Dear Editor.

Thank you for the high quality and intensive review of the manuscript and for the detailed comments and suggestions. We are glad to inform you that we have revised the manuscript judiciously based on each and every comment and suggestion provided. It is our sincerest belief that the scientific value has greatly increased as the result of this process and we now believe that it meets the PLOS ONE criteria. Please find below the point-by-point response to the comments and suggestions.

1. Data availability statement: You argue that “No - some restrictions will apply” regarding data availability but “All relevant data are within the manuscript and its Supporting Information files.”. Those two statements are not compatible. Please following [PLOS data policy](http://journals.plos.org/plosone/s/data-availability) provide the URL of the public repository and the conditions of access.

**Response:** The EPHIA data is not released yet, but will be available at this sites in 3 to 4 months. The dataset will then be uploaded on the PHIA website: <https://phia-data.icap.columbia.edu/>.

2. PLOS ONE being a multidisciplinary journal written for a general audience, the authors should not take for granted knowledge of what the 90-90-90 targets are as in line 68. That should be provided as background stating that the paper is concerned about the first 90. Instead, it does not to be fully described later in the results (l. 209).

**Response:** Agree. Defined the UNAIDS targets upfront in the first paragraph of the Introduction, and indicated that focus in this report is the first 90 in the last paragraph of the Introduction. The definition given in l. 209 is deleted.

3. A third concern is the way that unawareness is measured as a proportion of all HIV+. As the reviewer states, the policy implications of this research is to look for those unaware of their HIV+ status. While this focus is not wrong and corresponds to the first 90 target, it should go together with the complementary perspective: the proportion HIV+ unaware people among those declaring not to be HIV+, since this has implications regarding testing costs and cost-benefit of a testing programme. There is also no information regarding whether HIV+ unaware people had previously tested. Please provide at least descriptive measures in these respects, maybe a table similar to table 1 for those HIV+ unaware as proportion of those declaring to be HIV and some tabulation of those unaware HIV+ according to previous testing. This is a concern due to the effects that testing can have on behaviour, please check Gong, E. (2015), “HIV Testing and Risky Sexual Behaviour”. *Econ J*, 125: 32-60. <https://doi.org/10.1111/ecoj.12125>

**Response: U**nawareness is defined as a self-reported status among HIV-positive participants who either had never been tested for HIV or those who self-reported that their HIV status was negative. Those unaware HIV positives according to previous testing are included in Table 2 (under Sub-title: Ever been tested for HIV). Among those who were unaware of their HIV status, 84% had never been tested and 14% had been tested for HIV, a difference that was significant (p= 0.0001).

Please submit your revised manuscript by Jun 28 2021 11:59PM. If you will need more time than this to complete your revisions, please reply to this message or contact the journal office at [plosone@plos.org](mailto:plosone@plos.org). When you're ready to submit your revision, log on to <https://www.editorialmanager.com/pone/> and select the 'Submissions Needing Revision' folder to locate your manuscript file.

Please include the following items when submitting your revised manuscript:

- A rebuttal letter that responds to each point raised by the academic editor and reviewer(s). You should upload this letter as a separate file labeled 'Response to Reviewers'.

**Response:** Uploaded.

- A marked-up copy of your manuscript that highlights changes made to the original version. You should upload this as a separate file labeled 'Revised Manuscript with Track Changes'.

**Response:** Uploaded.

- An unmarked version of your revised paper without tracked changes. You should upload this as a separate file labeled 'Manuscript'.

**Response:** Uploaded.

If you would like to make changes to your financial disclosure, please include your updated statement in your cover letter. Guidelines for resubmitting your figure files are available below the reviewer comments at the end of this letter.

If applicable, we recommend that you deposit your laboratory protocols in [protocols.io](http://protocols.io/) to enhance the reproducibility of your results. Protocols.io assigns your protocol its own identifier (DOI) so that it can be cited independently in the future. For instructions see: <http://journals.plos.org/plosone/s/submission-guidelines#loc-laboratory-protocols>. Additionally, PLOS ONE offers an option for publishing peer-reviewed Lab Protocol articles, which describe protocols hosted on [protocols.io](http://protocols.io/). Read more information on sharing protocols at <https://plos.org/protocols?utm_medium=editorial-email&utm_source=authorletters&utm_campaign=protocols>.

Journal Requirements:

1) Please review your reference list to ensure that it is complete and correct. If you have cited papers that have been retracted, please include the rationale for doing so in the manuscript text, or remove these references and replace them with relevant current references. Any changes to the reference list should be mentioned in the rebuttal letter that accompanies your revised manuscript. If you need to cite a retracted article, indicate the article’s retracted status in the References list and also include a citation and full reference for the retraction notice.

**Response:** The reference list is edited; all references are double-checked and completed according to the ICMJE/ PLOS style.

The following changes are made in the References:

- References #1 and #2 in the previous version are removed, and replaced with references #1 and #2 in the revised version accommodating comments made by the Editor on the introduction.
- Reference #7 is replaced with a more relevant one.
- Reference #10 defining the Jackknife methods added per suggestion of the reviewer.
- Reference #12 is replaced with a more relevant one.

2) Please ensure that your manuscript meets PLOS ONE's style requirements, including those for file naming. The PLOS ONE style templates can be found at

<https://journals.plos.org/plosone/s/file?id=wjVg/PLOSOne_formatting_sample_main_body.pdf> and

<https://journals.plos.org/plosone/s/file?id=ba62/PLOSOne_formatting_sample_title_authors_affiliations.pdf>

**Response:** The manuscript is revised based on PLOS ONE's style requirements**.**

3) Please provide additional details regarding participant consent. In the ethics statement in the Methods and online submission information, please ensure that you have specified (1) whether consent was informed; (2) whether the ethics committee(s) approved digital documentation of consent; (3) whether parents/guardians provided written informed consent for minors to participate in the study, or whether the ethics committee(s) waived the need for this.

**Response:** Additional details are given on consent procedures, approved of the digital documentation of consent by the ethics committees, and written informed consent by parents/guardians for minors to participate in the study.

4)  Thank you for stating the following in the Acknowledgments Section of your manuscript:

[This project was conducted using the U.S. President’s Emergency Plan for AIDS Relief (PEPFAR) funds obtained though the U.S Center for Disease Control and Prevention (CDC) under the term of cooperative agreement #U2GGH001226. The findings and conclusions in this report are those of the authors and do not necessarily represent the official position of the funding agency..]

We note that you have provided funding information that is not currently declared in your Funding Statement. However, funding information should not appear in the Acknowledgments section or other areas of your manuscript. We will only publish funding information present in the Funding Statement section of the online submission form.

Please remove any funding-related text from the manuscript and let us know how you would like to update your Funding Statement. Currently, your Funding Statement reads as follows:

 [The funders had no role in study design, data collection and analysis, decision to
publish, or preparation of the manuscript.]

**Response:** Noted. The funding information declared in the manuscript is deleted. The amended statement is included in here as suggested.

This project was conducted using the U.S. President’s Emergency Plan for AIDS Relief (PEPFAR) funds obtained though the U.S Center for Disease Control and Prevention (CDC) under the term of cooperative agreement #U2GGH001226. The findings and conclusions in this report are those of the authors and do not necessarily represent the official position of the funding agency.

5) One of the noted authors is a group or consortium [EPHIA Study Team]. In addition to naming the author group, please list the individual authors and affiliations within this group in the acknowledgments section of your manuscript. Please also indicate clearly a lead author for this group along with a contact email address.

**Response:** The EPHIA Study Team is listed with affiliation for each member in the Ethiopia Population-based HIV Impact Assessment EPHIA 2017-2018 Final Report August 2020. pp 200-201. Available at: at:

<https://phia.icap.columbia.edu/wpcontent/uploads/2020/11/EPHIA_Report_280820_High-Res.pdf>.

The link is given in the Acknowledgement section along with the lead author and e-mail address.

[Note: HTML markup is below. Please do not edit.]

Reviewers' comments:

Reviewer's Responses to Questions

**Comments to the Author**

1. Is the manuscript technically sound, and do the data support the conclusions?

The manuscript must describe a technically sound piece of scientific research with data that supports the conclusions. Experiments must have been conducted rigorously, with appropriate controls, replication, and sample sizes. The conclusions must be drawn appropriately based on the data presented.

Reviewer #1: Yes

2. Has the statistical analysis been performed appropriately and rigorously?

Reviewer #1: Yes

3. Have the authors made all data underlying the findings in their manuscript fully available?

The [PLOS Data policy](http://www.plosone.org/static/policies.action#sharing) requires authors to make all data underlying the findings described in their manuscript fully available without restriction, with rare exception (please refer to the Data Availability Statement in the manuscript PDF file). The data should be provided as part of the manuscript or its supporting information, or deposited to a public repository. For example, in addition to summary statistics, the data points behind means, medians and variance measures should be available. If there are restrictions on publicly sharing data—e.g. participant privacy or use of data from a third party—those must be specified.

Reviewer #1: Yes

4. Is the manuscript presented in an intelligible fashion and written in standard English?

PLOS ONE does not copyedit accepted manuscripts, so the language in submitted articles must be clear, correct, and unambiguous. Any typographical or grammatical errors should be corrected at revision, so please note any specific errors here.

Reviewer #1: Yes

5. Review Comments to the Author

Please use the space provided to explain your answers to the questions above. You may also include additional comments for the author, including concerns about dual publication, research ethics, or publication ethics. (Please upload your review as an attachment if it exceeds 20,000 characters)

Reviewer #1: Reviewer Report for “Factors associated with Unawareness of HIV Positive Status in Urban Ethiopia: Evidence from the Ethiopia Population-based HIV Impact Assessment (2017-2018)”

Overall Comments:
The paper focuses on people who are HIV-positive and unaware of their status, broken down by different epidemiologic factors. Overall, this is an important study in guiding future testing and treatment efforts. In that vein, one of the strongest claims in the paper is that future efforts should focus on where there are more people living with HIV who are unaware of their status (Addis Ababa, Harari, Oromio), rather than where there is high prevalence (Gambella). However, the paper loses focus toward the end as the authors expound on every finding rather than only the main claims. There are several limitations to the study including that only urban enumeration areas which the authors state is 20% of the population, and possibly that ARV testing was only done for participants who reported being HIV-positive. Despite these limitations, the study uses national data to draw conclusions on how to increase HIV awareness. This paper makes an important contribution to the literature and help guide future HIV testing and treatment policies in Ethiopia.

Major Compulsory Revisions
Abstract:
Line 18: This line implies that all people who tested positive for HIV were also screened for ARVs, whereas later (Line 87), it is implied that only individuals who self-report HIV positive status were screened for ARVs. Please clarify.

**Response:** The statement in line 18 is correct (screening for ARVs was for all HIV-positive participants). Line 87 is edited (self-reported deleted and replaced by ….. on all participants who were HIV positive).

Also, throughout the paper, the terms “HIV positive,” “HIV positive status,” “HIV+,” “HIV infected,” and “PLHIV” are used. Unclear to me if this is intentional, but would benefit from consistency.

**Response:** Agree. Used HIV-positive and HIV-Positive status as appropriates across the manuscript, and HIV infection and transmission in a few places this is required

Results:
Line 188: I think ARV data could be a very important aspect of this study. Was ARV testing only done for participants who reported being HIV-positive? Readers would be very interested in the data on ARV testing among all HIV-positive individuals. It would change our interpretation of the positivity data as well as risk factors such as condom use.

**Response:** ARV was conducted for those who tested positive, not who reported HIV positive. Added ARV status among HIV-positives in Table 2.

Line 203: Please clarify the column headings in Table 2. Three of them are labeled “(N)” and it’s unclear what are the numerators and denominators for the “%” column.
Line 214: It’s unclear to me what the left y-axis of this graph is. I would assume it is percentage of people who tested HIV-positive who are unaware of their status, but these numbers are different from those in Table 2.

**Response:** Edited to clarify N and n. Table 2 is summarizes unawareness status by specific regions, and Figure 3 summarizes the burden of unawareness among all HIV positives across the regions.

Minor Essential Revisions
Abstract:
Line 23: Unclear to me why a percentage isn’t placed after the number tested HIV positive (3.2%). Is it due to the sampling strategy?

**Response:** No it is not due to the sampling strategy; 3.2% is now included after the number (614) in the results, both in the abstract and text.

Line 34: “One fifth of the urban population were” should be “One-fifth of the HIV-positive urban population were.”

**Response:** Yes, **c**orrected.

Introduction:
Lines 62-63: The first estimate of 75% refers to PLHIV knowing their HIV status, whereas the second numbers of 97% and 99% refer to knowledge of HIV. I’d consider either changing this to make the numbers more consistent or focus on framing each number to explain the train of logic.

**Response:** Revised the flow – Knowledge above HIV, then, awareness about HIV status. Order of references # 7 And #8 changed,

Line 68: You mention the “first 90 UNAIDS and country targets” but I don’t think the targets have been clearly introduced yet.

**Response:** Deleted from here as it is now defined upfront in the introduction (please also see response to academic editor’s comment #2 above)

Methods:
Line 77: Missing a comma between Afar and Amhara.

**Response:** Corrected.

Line 78: Do not need a comma between “Addis Ababa” and “and Dire Dawa”

**Response:** Deleted

Line 77-78: Consider using an m-dash (—) instead of colons given the use of two colons in the sentence.

**Response:** Done

Line 105: There is an extra space between “HIV” and “+”

**Response:** Edited; HIV-positive now.

Line 114: “Data was analyzed using” should be changed to “Data were analyzed using”

**Response:** Done

Line 122: Please clarify what is meant by “priority confounders.”

**Response:** Removed the word priority as it is not required.

Line 124: How was collinearity assessed?

**Response:** We assessed the collinearity of variables by looking at the variance inflation factor (VIF). This is now indicated in the manuscript.

Line 124: “was not significant on its” should be changed to “was not significant with its”

**Response:** Changed.

Line 125: Please clarify what is meant by “only the variable that improve the model was included.” I would assume that if two variables are collinear and one improves the model, then would both improve the model. Presumably one could improve the model more than the other.

**Response:** In this analysis, the process of variable selection, deleting, model fitting and refitting of the model was conducted many times. We have e checked each model for the goodness of fit (GOF). The variables that improved the GOF more than the others were included in the model. We have added “more than the other” to make this more specific

Results:
Line 145: Is there a reason no percentage is placed after the number HIV positive?

**Response:** No reason. Added.

Lines 146-147: “Among HIV positive participants, females constituted” should be changed to “Among HIV positive participants, 67.9% were females and 39% were”

**Response:** Edited.

Line 164: There is some inconsistent bolding in the table. The word “sex” in “Had no sex” is bolded. “N=606” is not bolded. Two words in “Did not have sex before age 15” appear bolded.

**Response:** Edited.

Line 166: The sub-heading “Self-reported HIV Status” does not have a colon at the end. Throughout the paper, some sub-headings have a colon and others do not.

**Response:** Edited based on PLOS style guidelines – font and consistently no colons.

Line 170: In Figure 1, is there a reason why Somali has a greater proportion that has tested and received results in the last 12 months than the proportion that ever tested and received results? I’m guessing this is due to incomplete data but should clarify.

**Response:** Figure 1 is for adult study participants irrespective of their HIV status. Revised Figure 1 and included only HIV positive adults ages 15-64.

Line 173: Why does this figure only include ages 25-64 when all others are 15-64?

**Response:** This is a typo. Corrected.

Line 177: “74% self-reported they were HIV” should be changed to “74% self-reported they were HIV-positive”

**Response:** Changed.

Line 178: “11% reported they had” should read “11% self-reported they had”

**Response:** Corrected.

Line 180: I think in general, the order of parentheses and brackets should be with parentheses on the outside and brackets on the inside. This is done multiple times throughout the paper.

**Response:** All edited making use of these consistent across the manuscript..

Line 189: “ARV status, HIV positive” should read “ARV status, among HIV positive”

**Response:** Edited.

Line 192: Some of the brackets/parentheses are mixed up in this line.

**Response:** Right. Edited.

Line 203: The last row presumably is the total among everyone. This row could be labeled more obviously or be closer to the top of the table.

**Response:** Last row labeled total.

Line 218: Table 3 actually shows quite a few other variables that are associated with unawareness of HIV-positive status in bivariate log binomial regression, for example certain regions and primary education. It’s possible that these were not included due to a correction for multiple hypothesis testing but it could be helpful to clarify why those positive results in the table are not included in the text.

**Response:** All variables that are associated with unawareness of HIV-positive status in the bivariate log binomial regression are now included in the description.

Line 225: It may help the reader to clarify that the Crude and Adjusted Prevalence Ratios are estimated using a log binomial model. For less statistically-inclined readers (including myself), there are a lot of terms in the methods that could be confusing.

**Response:** Crude and adjusted prevalence ratios are estimated using a log binomial model (this description is included in the methods). Jackknife method is defined and methods of collinearity assessment are described in the methods section.

Line 230: Why were these three variables controlled for? They were not the “priority confounders” as stated in line 121, nor are they significant variables in Table 2.

**Response:** They were not controlled. Sentence edited to correct this.

Line 232: the word “statistically” is unnecessary here as it already says “significantly”

**Response:** Agree. Deleted.

Line 233: Previously in the Results section, 95% CI were followed by a colon, but in these next two paragraphs, they are followed by a comma in some cases, colon in others, and no punctuation as well.

**Response:** Edited. Punctuations are now similarly used across the manuscript.

Discussion:
Line 246: one fifth should be “one-fifth” – applies throughout the paper.

**Response:** Corrected across the manuscript.

Line 263: “Kuwazulu Natal” should be “KwaZulu-Natal province”

**Response:** Corrected.

Line 287: There is a typo in “2030These and measures”

**Response:** Corrected.

Line 292: “door to door” should be hyphenated

**Response:** hyphenated.

Line 296: “contribute for the” should be “contribute to the”

**Response:** Corrected.

Line 317: “Voluntary Medical ale Circumcision” should be “Voluntary Medical Male Circumcision”

**Response:** Corrected.

Line 318: There is an extra period

**Response:** Deleted.

Line 327: “HIV positives participants” should be “HIV positive participants”

**Response:** Edited.

Line 350: “A previous study report indicated” should read “A previous study reported”

**Response:** Edited.

Line 353: “have a steady partner, and are not part” should read “have a steady partner, are not part”

**Response:** Edited.

Limitations:
Line 371: “Furthermore;” should read “Furthermore,”

**Response:** Edited.

Conclusions:
Line 379: There is an extra period at the end

**Response:** Removed.

I would add as one of the major conclusions that the number of unaware HIV+ individuals has a different distribution than the HIV prevalence or % unaware, which is a critical distinction to control the epidemic.

**Response:** Right. Added in the conclusion..

Discretionary Revisions

Abstract:
Lines 26-33: Could probably be shortened to something along the lines of: “In the bivariate analysis…, three regions (Oromia, Addis Ababa, and Harari), male gender, and young age (15-24 years) were significantly associated with HIV awareness of HIV status. In multivariate analysis, the same variables were associated with awareness of HIV status.”

**Response:** Agree. Shortened as suggested.

Line 31: The Adjusted Prevalence Ratios are actually risks and not odds. (Unless a prevalence odds ratio was used, which doesn’t appear to be the case)

**Response:** Yes, the prevalence ratio (PR) in this cross sectional study is analogous to the risk ratio (RR) of cohort studies. The section is revised based on comments

Introduction:
Lines 42-49: This paragraph jumps from discussing mortality (“mortality declined by 44%”) to infections (“infections decreased by 28%”), and then back to mortality (“The global decline in deaths from AIDS-related illness”). I would consider centering the introduction on the UNAIDS 2020 targets regarding awareness of HIV status.

**Response:** Agree. Revised centering the paragraph on the UNAIDS 2020 awareness target and also to improve the flow of the content.

Methods:
Line 116: May want to describe Jackknife replication. I’m unsure if it’s commonly used but readers may be more familiar with bootstrapping.

**Response:** Described and a reference (#11) added

Results:

Line 144: A table of characteristics of the general sample, including both HIV-positive and HIV-negative could be useful for comparison’s sake.

**Response:** Table revised to include info on total, positives and negatives

Lines 181-184: This sentence is a little unclear. May want to consider changing the wording to something along the lines of “In Afar, Addis Ababa, and Dire Dawa, more people never tested than self-reported negative, whereas in the rest of the other regions, more people self-reported negative.”

**Response:** Agree. Edited to make it clearer.

Line 206: Figure 3 seems to show in graphical representation the data in the first portion of Table 2. Is there a particular reason for the geographic representation of the unawareness? If not, can consider removing this figure.

**Response:** Agreed. Figure 3 deleted and related text edited.

Discussion:
Line 259: Please explain the overall capacity score.

**Response:** described.

Line 268: Just a note, this is exactly why the reader would be very interested in the ARV data!

**Response:** Yes. ARV data is now included.

Line 298: Although this is true regarding Gambella, as you stated in line 283, this would be a low-yield strategy.

**Response:** Agree. We have deleted the statement in line 298.

Line 308: The authors discuss the difference in transmission between PLHIV who are aware and unaware of their HIV status. They should consider a modeling analysis in the future!

**Response:** Yes, we will consider a modeling analysis in the future and this is indicated in the manuscript.

6. PLOS authors have the option to publish the peer review history of their article ([what does this mean?](https://journals.plos.org/plosone/s/editorial-and-peer-review-process#loc-peer-review-history)). If published, this will include your full peer review and any attached files.

If you choose “no”, your identity will remain anonymous but your review may still be made public.

**Do you want your identity to be public for this peer review?** For information about this choice, including consent withdrawal, please see our [Privacy Policy](https://www.plos.org/privacy-policy).

Reviewer #1: **Yes:** Roger Ying

[NOTE: If reviewer comments were submitted as an attachment file, they will be attached to this email and accessible via the submission site. Please log into your account, locate the manuscript record, and check for the action link "View Attachments". If this link does not appear, there are no attachment files.]

While revising your submission, please upload your figure files to the Preflight Analysis and Conversion Engine (PACE) digital diagnostic tool, <https://pacev2.apexcovantage.com/>. PACE helps ensure that figures meet PLOS requirements. To use PACE, you must first register as a user. Registration is free. Then, login and navigate to the UPLOAD tab, where you will find detailed instructions on how to use the tool. If you encounter any issues or have any questions when using PACE, please email PLOS at [figures@plos.org](mailto:figures@plos.org). Please note that Supporting Information files do not need this step.

*In compliance with data protection regulations, you may request that we remove your personal registration details at any time.* [*(Remove my information/details)*](https://www.editorialmanager.com/pone/login.asp?a=r)*. Please contact the publication office if you have any questions.*

|  | Reply Forward |
| --- | --- |
