# Supplementary material for: Factors associated with unawareness of HIV-positive status in urban Ethiopia: Evidence from the Ethiopia population-based HIV impact assessment 2017-2018
Source: PLoS One. 2021 Aug 11;16(8):e0255163. doi: 10.1371/journal.pone.0255163 (PMC8357455; doi:10.1371/journal.pone.0255163)
Supplement: Attachment — Submitted filename: Resubmission Comments.docx [file pone.0255163.s003.docx]

Resubmission Review for “Factors associated with unawareness of HIV-positive status in urban Ethiopia: Evidence from the Ethiopia Population-based HIV Impact Assessment (2017-2018)”

Thank you for addressing my prior concerns. My remaining comment is that the Discussion is quite long and unfocused. I have two suggestions for shortening. One is that comparisons to other studies can be shortened. For example, lines 255-256 are well summarized whereas lines 330-336 could be shortened without sacrificing information. The second suggestion is that the discussion can be more targeted. The discussion begins with a comparison of the unawareness rate in Ethiopia compared to other countries, and then hypothesizes several reasons why this is so. It then suggests why unawareness may be more important than prevalence. It then switches to geographic variations in testing strategies, then consequences of unawareness, geographic variation again, and then risky sexual activity. Although all sections are relevant and important, the reader would benefit from guidance regarding the most salient results rather than all.
